# Neuropeptide Kyotorphin (Tyrosyl-Arginine) has Decreased Levels in the Cerebro-Spinal Fluid of Alzheimer’s Disease Patients: Potential Diagnostic and Pharmacological Implications

**DOI:** 10.3389/fnagi.2013.00068

**Published:** 2013-10-30

**Authors:** Sara Matos Santos, Laura Garcia-Nimo, Sónia Sá Santos, Isaura Tavares, José A. Cocho, Miguel A. R. B. Castanho

**Affiliations:** ^1^Instituto de Medicina Molecular, Faculdade de Medicina da Universidade de Lisboa, Lisboa, Portugal; ^2^Laboratorio de Metabolopatías, Hospital Clínico Universitario de Santiago, Santiago de Compostela, Spain; ^3^Faculdade de Medicina do Porto, Departamento de Biologia Experimental, Porto, Portugal

**Keywords:** kyotorphin, pain, Alzheimer, drug, cerebro-spinal fluid, neuroprotection

## Abstract

In Alzheimer’s disease (AD), besides the characteristic deterioration of memory, studies also point to a higher pain tolerance in spite of sensibility preservation. A change in the normal tau protein phosphorylation is also characteristic of AD, which contributes to the pathogenesis of the disease and is useful in early diagnosis. Kyotorphin (KTP) is an endogenous analgesic dipeptide (Tyr-Arg) for which there is evidence of eventual neuroprotective and neuromodulatory properties. The objective of this work was to study the possible correlation between KTP and phosphorylated tau protein (p-tau) levels in cerebro-spinal fluid (CSF) samples of AD patients. CSF samples were collected from 25 AD patients and 13 age-matched controls (N), where p-tau and KTP levels were measured. We found a statistically significant difference between p-tau/KTP values in AD and N groups with an inverse correlation between p-tau and KTP values in AD samples. These results suggest that in the future KTP may be a candidate biomarker for neurodegeneration and may be a lead compound to be used pharmacologically for neuroprotection.

## Introduction

Medical and pharmacological developments in recent years have allowed a significant increase in aging of the population. Concomitant with the aging of the population the number of people with dementia has increased. Alzheimer’s Disease (AD) is the most prevalent neurodegenerative disease associated with dementia. It is a progressive debilitating disease with no known effective cure. Symptoms of memory loss begin at a variable age, usually between 40 and 65 years of age, progressing with over the years. It is expected that the current number of cases doubles by 2030, with a huge social and economic impact (Knickman and Snell, 2002).

Patients suffering from AD show a progressive deterioration of memory, orientation, emotional stability, speech, abstract thinking, motor skills, and ultimately self-care. The motor and cognitive deterioration in AD patients is accompanied by a reduction in the ability to communicate, which makes it difficult to detect pain in these patients. By failing to receive adequate pain treatment, structural and irreversible changes may occur in central systems structures involved in the transmission/modulation of nociceptive information, which accounts to chronic pain installation (Borsook, 2012). Curiously, the two components of the pain response are differentially affected in AD patients (Farrell et al., 1996; Benedetti et al., 1999). Whereas the sensory-discriminative component is preserved, pain tolerance, associated with the affective-emotional aspect, largely increases. These apparent discrepancies appear to have a neurobiological explanation since the somatosensory cortex and thalamic nuclei involved in sensory-discriminating component of pain response appear to be preserved in AD, while the neuronal loss was detected in the prefrontal and limbic structures, with obvious implications for and affective-emotional pain-related reactions (Benedetti et al., 1999; Borsook, 2012).

In pathophysiological terms, AD is characterized by cognitive losses, associated cholinergic deficit in the frontal brain area and extensive neuronal loss as well as synaptic changes in the neuronal pattern at the cortex and hippocampus. There is also evidence of neuronal tangle formation, made up of tau protein. Tau proteins are a group of neuronal microtubule-associated proteins, having a role in the regulation of neurite outgrowth, microtubule dynamics and axonal transport. There is strong evidence that in AD there is a change in the normal tau phosphorylation, which contributes to the pathogenesis of the disease. In fact, the abnormal phosphorylation leads firstly to a loss of function with decreased microtubule binding and, secondly, an increase of tau–tau interactions with negative effects on the neuronal stability (Johnson and Stoothoff, 2004). It has been well studied and commonly accepted that abnormally phosphorylated tau (p-tau) is the major protein subunit of Alzheimer’s paired helical filaments (PHFs), being useful in the early diagnosis of this pathology (Kurz et al., 1998). Tau is normally an intracellular protein, and the amount found in cerebro-spinal fluid (CSF) is low. The slow neurodegenerative process that occurs in AD leads to increased neuronal loss which may give rise to increased tau levels in CSF. CSF samples of AD patients show high p-tau levels compared to normal subjects, correlating with hyperphosphorylated tau in cortical brain biopsies (Seppälä et al., 2012).

The other neuropathological hallmark of the AD is the formation of amyloid plaques, made of amyloid-β (Aβ) peptide, derived from the amyloid precursor protein (APP). The “amyloid cascade hypothesis” states that an imbalance between the production and clearance of the Aβ is the initiating event in AD, with the increase in Aβ ultimately leading to tau pathology, neuronal degeneration, and dementia (Blennow et al., 2010). Aβ is produced during normal cell metabolism and secreted into the CSF and can also be used as a biomarker for AD, specifically its most abundant species – Aβ_1–42_, with AD patients consistently exhibiting a decrease of Aβ_1–42_ in CSF (Blennow, 2004; Blennow et al., 2010).

**Figure 1:**
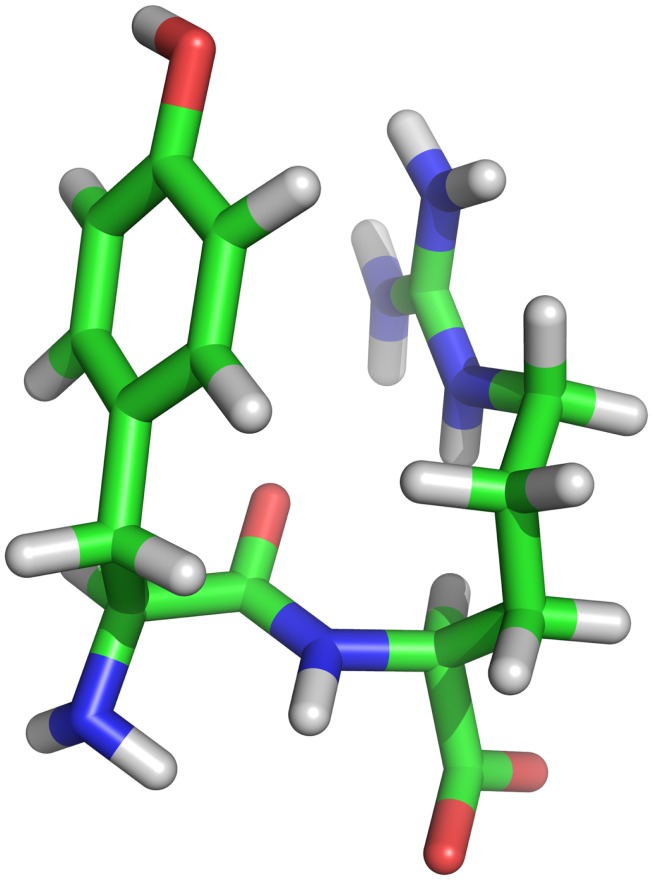
**Kyotorphin chemical structure (l-tyrosyl-l-arginine)**.

Kyotorphin (KTP) is a dipeptide (Tyr-Arg; Figure 1) with endogenous analgesic properties, first described by Takagi et al. (1979), isolated from bovine brain. It was subsequently isolated from other sources, including the brains of mice and rats (Ueda et al., 1980), guinea pig, rabbits, and squirrels (Ignat’ev et al., 1998) and detected in the CSF in humans (Nishimura et al., 1991). Despite being about fourfold more analgesic than endogenous opioids (Shiomi et al., 1981a), KTP effect is only induced after direct injection in the brain. This limited capacity to cross the blood-brain barrier prevents its pharmacological applications. KTP derivatives however have recently been demonstrated to be analgesic after systemic administration in several pain models which indicates the ability of KTP derivatives to cross the blood-brain barrier (Ribeiro et al., 2011a,b).

Kyotorphin is synthesized in nerve terminals and released by depolarizing stimuli (Shiomi et al., 1981b). It may modulate the synaptic transmission and directly excite cortical neurons. It was proposed that it exerts indirect opioid-like actions, producing analgesia through the release of met-enkephalin (Ribeiro et al., 2011b). Other studies in animal models have revealed the neuroprotective actions of KTP in the hippocampus and cerebellum (Nazarenko et al., 1999; Bocheva and Dzambazova-Maximova, 2004). In addition, there is further evidence that the peptide has neuroprotective and neuromodulating properties (Santalova et al., 2004; Dzambazova and Bocheva, 2010), also acting as a neuroleptic and inhibiting calcium-dependent currents in the post-synaptic membrane (Gorenkova et al., 2002; Santalova et al., 2004). There is also an anticonvulsant effect reported in animal models of epilepsy (Godlevsky et al., 1995; Shandra, 1999).

Although there is no immunohistochemical mapping of kyotorphin-containing neurons in the brain, rat brain homogenates show that the highest levels are found in the midbrain, pons, and medulla oblongata, correlating with the areas most sensitive to morphine or electrical stimulation-induced analgesia. However when the results are expressed as a percentage of the total KTP found in different sections, the cortex seems to contain around 50%, an area where enkephalins receptors are low, which suggests that KTP non-opioid actions are important in its neurochemical action (Ueda et al., 1982; Dzambazova and Bocheva, 2010). For KTP, a non-opioid activity was also described in the peripheral nervous system and other organs like the heart (Inoue et al., 1997; Li et al., 2006). It was shown that the KTP, due to its l-Arginine residue, could act as substrate for nNOS [nitric oxide synthase (NOS), located in neurons] (Arima et al., 1996, 1997). Moreover, in recent years, there is increasing evidence that AD may be primarily a vascular disease with neurodegenerative consequences, rather than a neurodegenerative disorder with vascular consequences (de la Torre and Stefano, 2000). Two key factors for the development of AD have been proposed: aging and decreased cerebral perfusion. The convergence of them will result in the so-called “critically attained threshold of cerebral hypoperfusion” (CATCH). It was proposed that the CATCH leads to a distortion of the architecture of the brain capillary, involving impairment of the release of nitric oxide (NO), which will affect the signaling between the immune, nervous, and cardiovascular systems. Tissues with decreased levels of NO may be severely deregulated. The concomitance of advanced age and risk factors may contribute to vascular lesions involving eNOS (located in the endothelium), causing a deficit of NO to such a degree that this could initiate the neurodegenerative changes characteristic of AD (de la Torre and Stefano, 2000).

So it seems that KTP could function in two separate but correlated domains: pain and neuromodulation. This assumption would make us pose another question: when neuronal death occurs, will there be a loss of KTP production that could worsen neurodegeneration and with concomitant alteration of pain threshold in patients? This would place KTP as a central player in the amplification of the continuous degradation of the clinical condition of AD patients. The goal of the present work was to study the possible correlation between KTP and p-tau levels in CSF samples of Alzheimer patients to contribute to a better understanding of KTP mechanisms of action and open new horizons in this area of research.

## Materials and Methods

All participants and caregivers were informed about the aim and procedure of this work and gave their oral and written informed consent prior to the commencement of the study.

We evaluated 25 patients with the diagnosis of AD, followed in Neurology consultation at a Portuguese Hospital with moderate cognitive impairment (stage 5 of the Global Deterioration Scale), and 13 age and sex-matched controls. The control group comprised healthy patients, who underwent lumbar puncture because of suspected (but not confirmed) subarachnoid hemorrhage or other indications in the usual neurological survey. Subjects with a history of cerebral trauma, transient ischemic attack, neoplasm, epilepsy, disturbances of consciousness, or focal brain disorders were excluded from participation in this study. Traumatic spinal punctures (i.e., with evidence of blood contamination) were also excluded from the study.

After lumbar puncture, the CSF samples were collected in polyethylene tubes, protected from light exposure with aluminum foil and stored at −80°C until analysis (without additional freeze-thaw cycles). Appropriate evaluation of the degree of patient’s dementia was performed by quantifying p-tau in CSF with a commercial tau-specific sandwich ELISA kit (Innogenetics, Ghent, Belgium).

Due to low levels of Kyotorphin that are estimated to be present in the CSF (10^−9^ M) (Nishimura et al., 1991) it was necessary to resort to advanced analytical techniques namely electrospray ionization tandem mass spectrometry (ESI – MS/MS). The specimens of CSF were dried under a stream of nitrogen, derivatized with HCl-butanol and reconstituted with a solution containing CH_3_CN/H_2_O 7:3 and 15 mL/L formic acid. This procedure it is similar to the routine method is used in our laboratory for metabolite profiles in urine samples (Rebollido-Fernandez et al., 2012). The solution was shaken on a vortex-mix system for 15 min at room temperature, and 20 μL solution was injected into the mass spectrometer. Calibration solutions were prepared with different additions of KTP in a CSF matrix, were at concentrations from 0.625 to 10 nM (Figure S1 in Supplementary Material). We obtained a detection limit of 0.8 nM. The recovery ranges between were 60.7 and 100.9%, at the working levels of 2.5 nM was 85.4%. The precision ranges between were 1.9 and 14.8%. We used an API 4000 triple quadrupole mass spectrometry (Sciex Applied Biosystems) equipped with an electrospray source with the turbo gas temperature set at 750°C. The equipment was operated in positive ionization polarity at a potential of 5400 V. We performed multiple reaction monitoring (MRM) measurements, with Q1 394.3 and Q3 136.1 (Figure S2 in Supplementary Material).

The results are expressed as mean ± SD unless otherwise stated; parametric data were analyzed with the Student’s *t*-test and *p* < 0.05 was considered statistically significant. Statistical analysis was performed using the SPSS software version 11.5.1 (2002, SPSS base 11.0 for Windows SPSS Inc., Chicago, IL, USA).

## Results

In the AD group 63% of individuals were female and 37% male, with an average age of 70.1 ± 8 years. The mean time of diagnosis and follow-up in neurology consultation was of 16 months. These patients were classified in clinical grounds as having AD in moderate stage, with mini-mental state examination (MMSE) values of 14.3 ± 6. In the N group, patients had the same population characteristics but without any known neurodegenerative disease (Table 1).

**Table 1:** **Alzheimer’s disease patient characterization and CSF concentration results (mean ± SD; SE, standard error)**.

	AD group	N group	*p*-Value
Male/female (%)	63/37	56/44	–
Age (years)	70 ± 8	68 ± 6	>0.01
KTP (nM)	1.8 ± 0.6 (SE 0.1)	3.4 ± 1.2 (SE 0.3)	<0.01
p-Tau (pg/mL)	403.2 ± 157.6 (SE 31.5)	68.8 ± 18.3 (SE 5.2)	<0.01

We found a statistically significant difference (*p* < 0.01) between p-tau/KTP values in AD and N groups (Table 1). Regarding p-tau in AD group the values were 403.2 ± 157.6 pg/mL whereas in the N group the values were 68.8 ± 18.3 pg/mL (*p* < 0.01). Regarding KTP in AD and N groups the values were 1.8 ± 0.62 and 3.4 ± 1.2 nM, respectively.

**Figure 2:**
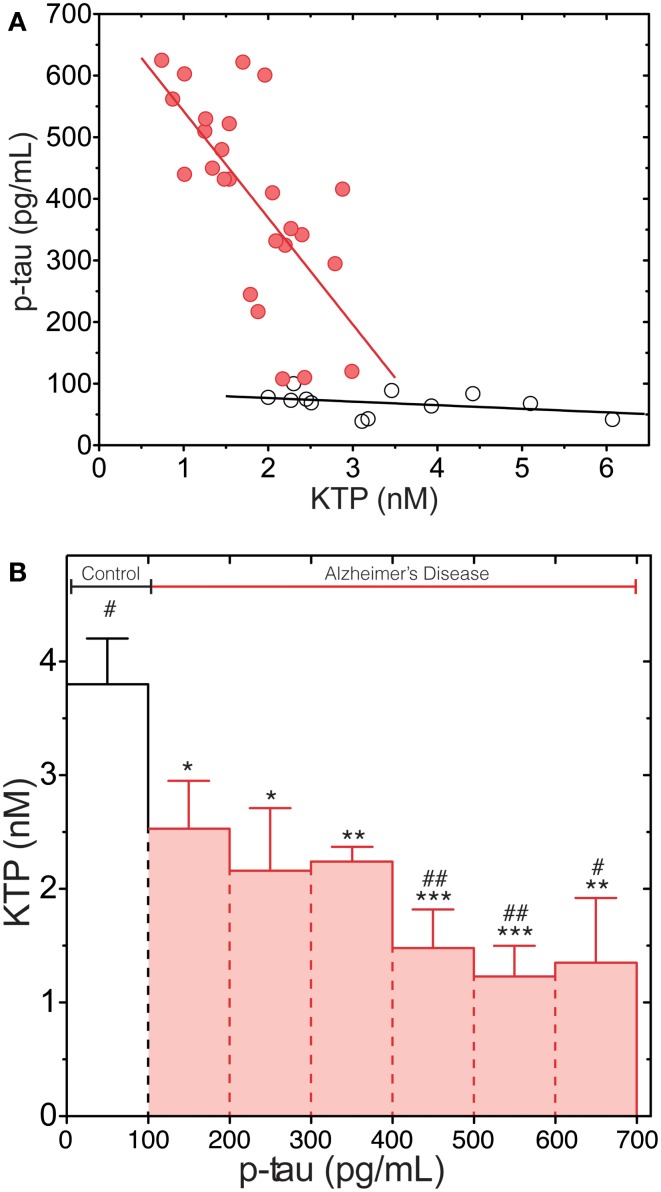
**p-Tau levels dependence on KTP concentration both in AD (red) and N (white) group**. **(A)** Complete data set with individual values. A linear regression line (least squares method) is plotted as a guide to the eye. **(B)** The data set presented in **(A)** was clustered in classes according to regular intervals of amplitude of 100 pg/mL of p-tau concentration and averaged for KTP concentration (all N group data are in the 1- to 100-pg/mL p-tau interval and AD values are in classes above 100 pg/mL; error bars represent standard error). The average KTP value of all classes was compared to control (*p < 0.04, **p < 0.0075, ***p < 0.0006) or the 100- to 200-pg/mL p-tau class (^#^*p* < 00.04, ^##^*p* < 0.0046) using the one-way ANOVA with Dunnett’s post-test.

Figure 2A shows a plot of the p-tau and KTP concentrations in the CSF of AD patients and control group (N). There is a clear inverse correlation between the levels of p-tau and KTP in the AD group (Pearson correlation coefficient of −0.69) in contrast with the N group (Pearson correlation coefficient of −0.38), This is corroborated by the application of a linear regression model to fit the data: the slope is not significantly different from zero in the N group (*p* = 0.22). The data was also clustered and the average KTP level was plotted against the concentration of p-tau for different classes (Figure 2B). This plot confirms that all data points in N are in the lowest class of p-tau level while simultaneously having the highest average KTP level. All AD classes have significantly lower levels of KTP than the control group, N. In AD patients decreasing average KTP levels are concomitant with increasing p-tau levels. The statistical analysis shows that above 400 pg/mL all AD classes have significantly lower KTP levels than the class 100–200 pg/mL. This result further supports an inverse relationship between KTP and tau in the CSF of AD patients.

## Discussion

Our study is in agreement with previous studies aiming at other neuromolecules (Sulkava et al., 1985; Blennow et al., 2010) that have shown that in AD and vascular dementia, as the disease progresses several neuropeptides levels fall. Here we also found a statistically significant difference in KTP levels between AD patients and N individuals, being lower in the first. This can be explained by to a disease-specific cortical thinning and hippocampal volume loss, with an acceleration phase during the early stages of the AD (Sabuncu et al., 2011), meaning less cortical mass, which possibly results in less KTP production capability and KTP dropping levels in the CSF of these patients (Figure 2). Moreover, there is an inverse correlation between p-tau and KTP in CSF in AD patients. p-Tau in CSF acts as a marker of neurodegeneration, being released from senescent neurons. KTP is also produced in neuronal cells, its levels naturally falling as neurons die. As more neuronal cells are destroyed in the course of the disease p-tau is released and KTP production is impaired (Figure 2).

Lower levels of a molecule with analgesic properties such as KTP may help to explain why AD patient population is believed to have an increased incidence of hidden chronic pain (see Introduction); in agreement, previous studies (Nishimura et al., 1991) have shown that KTP levels decrease in chronic pain conditions. Another potential implication of these results stems from the correlation between KTP and NO production in AD patients as lower levels of KTP might lead to a decreased NOS activity with consequent fall of NO levels. This NO deficit and consequent cerebral hypoperfusion (see Introduction) compromise further or even help to initiate neurodegenerative deregulations characteristic of AD, with increased both neuronal loss and levels of p-tau.

### Potential implications

One important implication of the results here presented is that KTP is a candidate biomarker for neurodegeneration. Several studies in animal models (Takagi et al., 1979; Nazarenko et al., 1999; Santalova et al., 2004; Ribeiro et al., 2011b) have shown that KTP functions not only as a potent endogenous analgesic molecule but also as neuroprotector and neuromodulator. We suggest further studies in animal models in order to unveil if exogenous administration of KTP (with increase of its CSF levels) can result in neuroprotection. There is evidence of the KTP role in integrative functions in animals, particularly in the field of exploratory activity (Kolaeva et al., 2000), which are not inhibited by naloxone, therefore independent of its analgesic effects. It is also important to recall that amidated KTP and other KTP-related molecules have important analgesic efficacy, as shown in previous studies (Ribeiro et al., 2011a,b). A dual neuroprotective analgesic action in a single drug would be of utmost importance.

**Figure 3:**
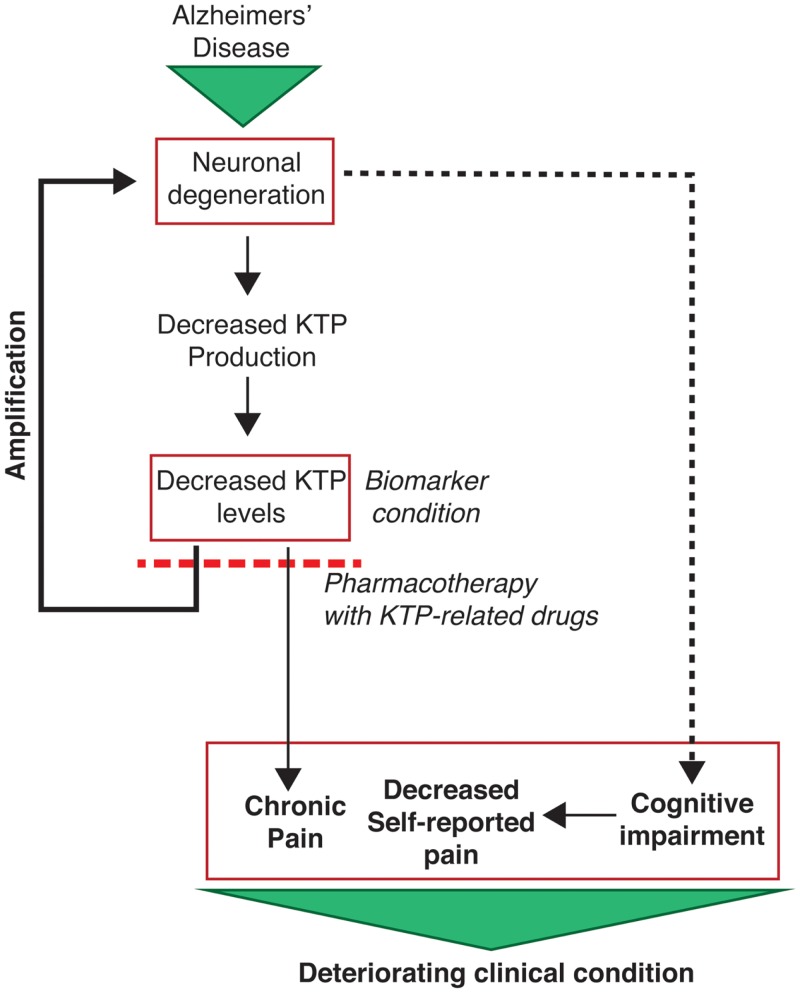
**Outline of the potential clinical implications of the results of the present study**. Neuronal degeneration (increased p-tau levels) leads to decreased KTP production and account to the detected decreased KTP levels, which in turn causes a positive feedback loop potentiating neuronal degeneration. Concomitantly, decreased KTP levels cause changes in affective pain tolerance which may account to chronic pain installation. This is particularly relevant as AD patients have limited capacity to report their pain due to their cognitive impairments. Altogether, these conditions set the severity of the clinical conditions of AD patients. Hope arises from the potential use of KTP-related drugs with dual neuroprotective and analgesic action, the latter being demonstrated for amidated kyotorphin (Ribeiro et al., 2011a) and a tandem ibuprofen-KTP drug (Ribeiro et al., 2011b). In addition, a decreased level of KTP in the CSF is a potential additional biomarker for AD.

In summary, this study points to KTP as a possible but overlooked biomarker with potential clinical dual importance in the context of pain and neurodegeneration in Alzheimer disease (Figure 3).

## Conflict of Interest Statement

The authors declare that the research was conducted in the absence of any commercial or financial relationships that could be construed as a potential conflict of interest.

## Supplementary Material

The Supplementary Material for this article can be found online at http://www.frontiersin.org/Aging_Neuroscience/10.3389/fnagi.2013.00068/abstract

Figure S1 **Calibration curve of KTP (kyotorphin) in a CSF matrix (0.625–10 nM)**.

Figure S2 **Multiple reaction monitoring (MRM) measurements, with Q1 394.3 and Q3 136.1, using an API 4000 triple quadrupole mass spectrometry (Sciex Applied Biosystems) equipped with an electrospray source**.
