# Supplementary figures and images for: Neuropeptide Kyotorphin (Tyrosyl-Arginine) has Decreased Levels in the Cerebro-Spinal Fluid of Alzheimer’s Disease Patients: Potential Diagnostic and Pharmacological Implications
Source: Front Aging Neurosci. 2013 Oct 30;5:68. doi: 10.3389/fnagi.2013.00068 (PMC3812564; doi:10.3389/fnagi.2013.00068)

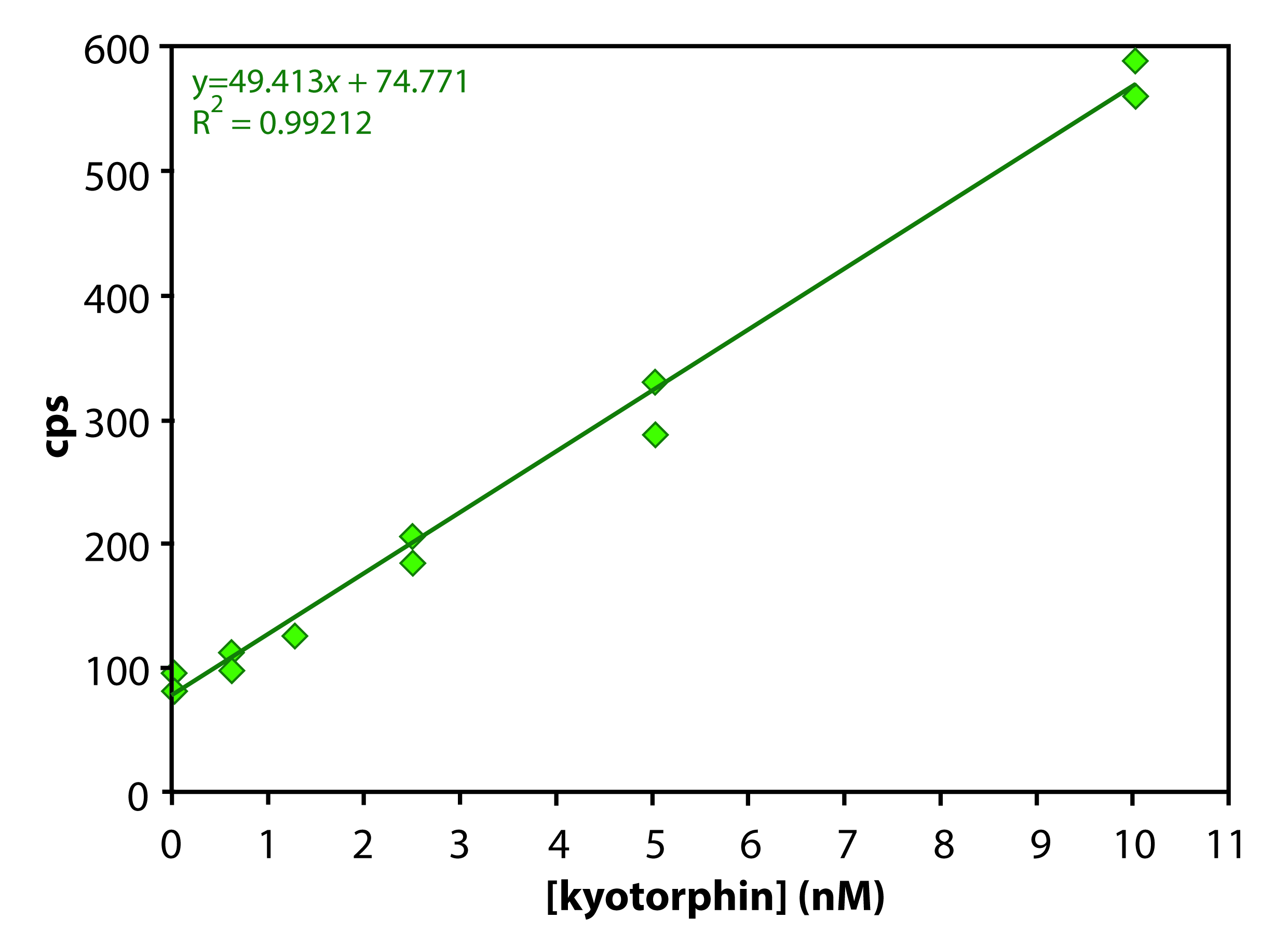

Supplement: Figure S1 — Calibration curve of KTP (kyotorphin) in a CSF matrix (0.625–10 nM). [file 66158_Castanho_Presentation1.ZIP › Figure S1.tif]

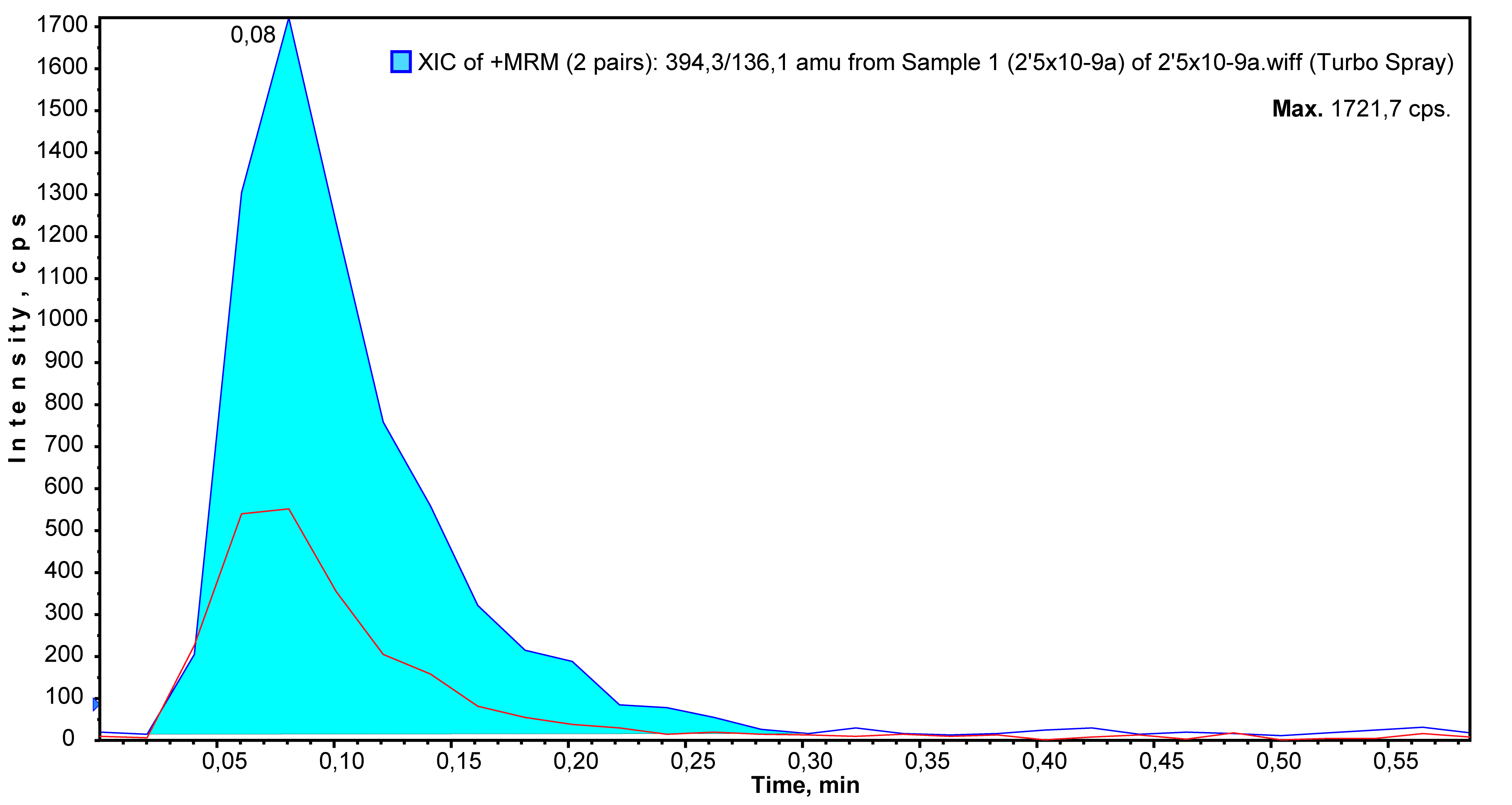

Supplement: Figure S1 — Calibration curve of KTP (kyotorphin) in a CSF matrix (0.625–10 nM). [file 66158_Castanho_Presentation1.ZIP › Figure S2.tif]
